# Molecular characterization of carbapenem resistance mechanisms and phenotypic correlations in clinical *Klebsiella pneumoniae* isolates from Ningbo, China

**DOI:** 10.3389/fmicb.2025.1546805

**Published:** 2025-05-14

**Authors:** Xuedan Qiu, Min Jiang, Jianqiang Xu, Qiaoping Wu, Chenyao Lin, Weiying Li, Qingcao Li

**Affiliations:** ^1^Department of Clinical Laboratory, The Affiliated Li Huili Hospital of Ningbo University, Ningbo, China; ^2^Department of Clinical Laboratory, Langxia Street Health Service Center, Ningbo, China

**Keywords:** *Klebsiella pneumoniae*, carbapenem-resistant, antibacterial susceptibility, resistance genes, MLST

## Abstract

**Objective:**

The purpose of this study is to understand the antimicrobial susceptibility and molecular distribution characteristics of carbapenem-resistant *Klebsiella pneumoniae* (CRKP) in the region, and to evaluate their correlation. Additionally, the study aims to investigate the transmission status of these strains.

**Methods:**

A total of 150 CRKP collected from January 2019 to December 2021 in the Ningbo region were included in this study. Antimicrobial susceptibility testing was performed using broth microdilution method following CLSI guidelines (CLSI, 2023). The tested agents included: (1) basic antimicrobials (tigecycline, polymyxin B, ceftazidime-avibactam); and (2) combination therapy candidates (ertapenem, imipenem, levofloxacin, piperacillin-tazobactam, ceftriaxone, cefepime, trimethoprim-sulfamethoxazole, fosfomycin, amikacin, aztreonam, chloramphenicol, amoxicillin-clavulanate, ceftazidime). Resistance genes were detected using polymerase chain reaction (PCR). Multi-locus sequence typing (MLST) was employed to analyze the molecular characteristics and evolutionary trends of the strains to determine their clonal relationships.

**Results:**

The 150 strains of CRKP exhibit high resistance rates to various conventional drugs; The sensitivity rates to tigecycline, polymyxin B, and ceftazidime-avibactam were 98.7, 98.0, and 68%, respectively; Conversely, the sensitivity rates to fosfomycin, amikacin, and chloramphenicol were 72.0, 40.0, and 16.7%, respectively; The main proportions of carbapemen genes producing in CRKP are as follows: *KPC-2* (61.3%), *NDM-5* (14.7%), *IMP-4* (8.0%), *OXA-232* (6.0%), and *OXA-181* (1.3%); The main proportions of β-lactamase resistance genes are as follows: *CTX-M-1* (13.33%), *CTX-M-3* (25.33%)*, CTX-M-9* (17.33%)*, CTX-M-14* (34.67%)*, SHV-1* (26.66%), *SHV-11* (66.66%), *SHV-12* (18.66%), and *SHV-28* (10.00%); CRKP carrying class A, B, and D carbapenemases had a sensitivity rate greater than 96% for tigecycline and polymyxin B, while their sensitivities to ceftazidime-avibactam, aztreonam, and amikacin varied significantly (*p* < 0.01). Analysis of the MLST results for CRKP revealed that ST11 strains were predominant in the region. There was a significant difference in the resistance genes carried by ST11 strains compared to non-ST11 strains. While different healthcare institutions exhibited variations in ST types, the strains generally showed high homogeneity.

**Conclusion:**

In the region, CRKP showed high sensitivity to tigecycline, polymyxin B, ceftazidime-avibactam, fosfomycin, amikacin, and chloramphenicol. The main carbapenemase genes identified were *KPC-2* and *NDM-5*. The inhibitory effects of ceftazidime-avibactam, aztreonam, and amikacin varied for CRKP carrying different enzyme types. ST11 strains were predominant in the region. There was a significant difference in the resistance genes carried by ST11 strains compared to non-ST11 strains. Clonal dissemination was observed both within the same healthcare institution and between different institutions.

## 1 Introduction

*Klebsiella pneumoniae* (KP) commonly colonizes the human nasopharynx, skin, and intestines and is one of the main pathogens causing hospital-acquired infections. It poses significant risks and can lead to multiple systemic infections such as meningitis, pneumonia, abdominal infections, and bloodstream infections (Jiang et al., 2024). In recent years, the emergence of carbapenem-resistant *Klebsiella pneumoniae* (CRKP) has become a significant public health concern, particularly in China. The widespread use of carbapenem antibiotics has led to a steady increase in CRKP detection rates (Hu et al., 2020). According to data from the China Antimicrobial Resistance Surveillance System (CARSS, 2014–2023),^1^ the prevalence of CRKP has shown a consistent upward trend in Zhejiang Province, with the Ningbo region being particularly affected (Gao et al., 2024). In healthcare settings in Ningbo, CRKP infections have exhibited a marked increase, reflecting a growing challenge in the management of antimicrobial resistance in the area. CRKP possesses significant pathogenicity, with mortality rates for bloodstream infections caused by it reaching as high as 45–75% (Venugopalan et al., 2017; Wang et al., 2018; Soares de Moraes et al., 2022). CRKP exhibits high resistance to commonly used antimicrobial drugs in clinical practice, resulting in a gradual reduction in available sensitive medications. Its broad transmission routes significantly increase the difficulty of treatment. In recent years, experts (Rodríguez-Baño et al., 2015; Guan et al., 2016) both domestically and internationally have reported reaching a consensus recommending a combination therapy approach for the treatment of CRKP infections. This approach is based on a combination of tigecycline, polymyxin B, imipenem, and ceftazidime-avibactam. Additional drugs included in the combination may be aminoglycosides, fosfomycin, and amoxicillin-clavulanic acid. Indeed, there are discrepancies in the reported sensitivity of these foundational antimicrobial agents for multidrug-resistant organisms and combination therapy drugs, including CRKP. Currently, there is a lack of systematic testing and evaluation in this regard. The mechanism of carbapenem resistance in CRKP is complex, with the most common being the production of carbapenemases (Sarva et al., 2023). Currently, carbapenemases are classified into three classes, A, B, and D, according to the Ambler classification system. Class A enzymes are mainly represented by *KPC* and *GES*, Class B enzymes are mainly represented by *IMP, VIM, GIM, SPM, SIM*, and *NDM,* and Class D enzymes are mainly represented by *OXA-48* (Çalık et al., 2022). The distribution of these carbapenemase genes varies significantly among CRKP strains across different regions (Angles-Yanqui et al., 2020; García-Betancur et al., 2021; Ge et al., 2024; Guo et al., 2024). In China, among CRKP clinical isolates, the primary carbapenemase types are *KPC* and *NDM*, with a small proportion of strains carrying *OXA-48* or *IMP-type* carbapenemase genes (Han et al., 2020; Li et al., 2021). Notably, several studies have reported that KPC-2 and NDM-1 are the most prevalent carbapenemases among CRKP clinical isolates in Eastern China, including the Ningbo region (Ding et al., 2024; Zhao et al., 2021), reflecting both regional epidemiological characteristics. Furthermore, strains producing different types of enzymes exhibit significant differences in resistance characteristics. The sensitivity of CRKP carrying different carbapenemase genes to antimicrobial drugs also varies, thereby affecting drug selection. Clearly identifying the distribution of carbapenemase types among CRKP strains in the local region is crucial for various aspects, including early intervention in CRKP infections, selecting empiric therapy, and enhancing treatment success rates. This study aims to understand the corelation between molecular and phenotype of CRKP in the Ningbo region, and to evaluate their correlation to lay a foundation for the treatment of CRKP infections. Additionally, it seeks to map the dissemination patterns of these strains, providing a scientific basis for the prevention and control of hospital-acquired infections in the region. By integrating epidemiological data with molecular insights, this research will contribute to the development of targeted interventions and improved management strategies for CRKP infections in healthcare settings.

## 2 Materials and methods

### 2.1 Bacterial strains and specimen source

In this study, a total of 150 strains of CRKP were selected from multiple hospitals located in Ningbo, Zhejiang Province, China, from January 2019 to December 2021. The hospitals included Hospital A (with two campuses: Campus I and Campus II), Hospital B, Hospital C, Hospital D, and Hospital E. The distribution of strains across hospitals, patient age, gender and departments was analyzed. This study was approved by the Ethics Committee of Ningbo Medical Centre Lihuili Hospital, Ningbo University (KY2023SL347-01). The specimen types included sputum, bronchoalveolar lavage fluid, urine, blood, puncture fluid, drainage fluid, pleural/peritoneal fluid, and bile, and strain identification was conducted using a mass spectrometer (Zhongyuan Huji, China). *Escherichia coli* ATCC 25922 served as the quality control strain for strain identification and antimicrobial susceptibility testing, and purchased from the National Center for Clinical Laboratories, Ministry of Health.

### 2.2 Drug susceptibility test

Antimicrobial susceptibility testing was performed using the broth microdilution method following CLSI guidelines (CLSI 2023). Briefly, bacterial suspensions were adjusted to 0.5 McFarland standard and diluted 1:200 in cation-adjusted Mueller-Hinton broth, with 100 μL aliquots dispensed into microdilution plates containing graded antimicrobial concentrations. After incubation at 35°C for 16–20 h, the minimum inhibitory concentration (MIC) was determined as the lowest concentration showing complete growth inhibition. The susceptibility results for ertapenem, imipenem, levofloxacin, piperacillin-tazobactam, ceftriaxone, cefepime, trimethoprim-sulfamethoxazole, ceftazidime-avibactam, amoxicillin-clavulanic acid, fosfomycin, ceftazidime, aztreonam, amikacin, and chloramphenicol were interpreted according to Clinical and Laboratory Standards Institute (CLSI) 2023 standards. Polymyxin B susceptibility was evaluated using European Committee on Antimicrobial Susceptibility Testing (EUCAST) version 10.0 criteria, while tigecycline breakpoints were determined based on standards from both the U.S. Food and Drug Administration (FDA) and China’s National Medical Products Administration (NMPA).

### 2.3 Screening of antibiotic resistance genes and whole-genome sequencing

In order to identify the presence of carbapenemase resistance genes, we conducted PCR amplification using specific primers targeting *KPC-2, NDM-1, NDM-5, VIM, IMP-1, IMP-2, IMP-4, OXA-232, OXA-181, IMI, SME, GES, GIM, SIM, SPM, AIM*, and *DIM*. These primers, designed to detect markers for carbapenemase resistance genes (refer to Supplementary Table 1), were utilized to screen for the presence of these genes in the template DNA of bacterial isolates. The PCR mixture consisted of a total volume of 25 μL, comprising 1 μL of genomic DNA template, 1 μL of each primer, 12.5 μL of Premix-rTaq PCR solution (manufactured by TaKaRa, Japan), and 9.5 μL of distilled water. The PCR procedure was conducted utilizing an ABI Veriti Thermal Cycler (Applied Biosystems, Singapore). The template was initially subjected to denaturation at a temperature of 94°C for a duration of 5 min. This was followed by 30 cycles consisting of denaturation at 94°C for 45 s, annealing at 55°C for 45 s, and extension at 72°C for 1 min. A final extension step was performed at 72°C for 10 min. The reaction conditions for β-lactamase genes (*DHA, CIT, EBC, MOX, ACC, FOX, CMY, TEM, SHV, CTX-M-1, CTX-M-2, CTX-M-3, CTX-M-8, CTX-M-9, CTX-M-10, CTX-M-14*, and *CTX-M-25*), outer membrane protein genes (*OmpK35* and *OmpK36*), and efflux pump genes (*acrA, oqxB, kexD, kdeA, kpnE, emrB, oqxA*, and *qacEΔ1*) may vary slightly. The PCR products were subsequently confirmed through electrophoresis and sequencing. The presence of carbapenemase genes was confirmed by aligning assembled contigs against the CARD database using BLASTn (Alcock et al., 2020). The primer sequences for other antibiotic resistance genes are provided in Supplementary Table 1, which were used to screen whether these genes are present in the template DNA of bacterial isolates. Due to limited funds, we selected 47 strains of CRKP from a total of 150 isolates for whole genome sequencing (WGS) using next-generation sequencing (NGS). The selection criteria were based on the following factors: (1) the distribution of resistance genes, particularly carbapenem resistance genes, to ensure that all major resistance genotypes were represented; (2) antimicrobial susceptibility profiles, with strains exhibiting diverse resistance patterns prioritized to capture the full spectrum of resistance mechanisms; and (3) the epidemiological distribution of strains across hospitals, patient demographics (age, gender), and clinical departments (e.g., ICU, respiratory, surgery). Genomic DNA was extracted and sent to Novogene (Beijing Novogene Bioinformatics Co., Ltd., Beijing, China) for WGS, which was performed using the Illumina HiSeq 4000 platform (Illumina, San Diego, CA, United States). Raw sequencing data obtained from the Illumina HiSeq 4000 platform were subjected to quality control using FastQC (v0.11.9) to assess read quality (de Sena Brandine and Smith, 2019). Low-quality reads and adapters were trimmed using Trimmomatic (v0.39) with the following parameters: SLIDINGWINDOW:4:20 and MINLEN:50 (Bolger et al., 2014). Clean reads were then assembled *de novo* using SPAdes (v3.15.4) with default parameters to generate draft genomes (Bankevich et al., 2012). The quality of the assemblies was evaluated using QUAST (v5.0.2) (Gurevich et al., 2013).

### 2.4 Determination of phylogenetic groups of *Klebsiella pneumoniae* by MLST

MLST analysis was performed to determine the sequence types (STs) of 150 CRKP isolates by amplifying seven housekeeping genes (gapA, infB, mdh, pgi, phoE, rpoB, and tonB). The sequences were compared with the *Klebsiella pneumoniae* MLST database (Jolley et al., 2018)^2^ to assign STs. A minimum spanning tree (MST) was constructed using PHYLOViZ Online (Nascimento et al., 2017)^3^ by uploading FASTA files containing housekeeping gene sequences and strain metadata. The MST was visualized with adjustments to node size, color, and other parameters.

### 2.5 Statistical analysis

Statistical analyses were performed using SPSS 26.0. Differences in resistance gene distribution and antimicrobial susceptibility profiles among ST types and carbapenemase gene carriers were evaluated using the chi-square test or Fisher’s exact test, with a significance level of *p* < 0.01.

### 2.6 Data availability

The complete genome sequences of 47 strains of CRKP were deposited in GenBank with accession numbers PRJNA1241480.

## 3 Results

### 3.1 Analysis of strain origin

The distribution of CRKP strains across hospitals revealed that Hospital A Campus I accounted for the highest proportion of cases (36.7%, 55/150), followed by Hospital A Campus II (24.6%, 37/150), Hospital B (14.0%, 21/150), Hospital C (10.7%, 16/150), Hospital D (8.0%, 12/150), and Hospital E (6.0%, 9/150). In terms of patient demographics, the majority of CRKP infections occurred in individuals aged 55 years or older, accounting for 72.0% (108/150) of the cases. The median age of patients was 65 years, with an age range of 18 to 92 years. Gender distribution was relatively balanced, with male patients representing 52.0% (78/150) and female patients representing 48.0% (72/150) of the cases. Regarding departmental distribution, the intensive care unit (ICU) had the highest proportion of CRKP cases, representing 30.0% (45/150) of the isolates. This was followed by the respiratory department (23.3%, 35/150), general surgery (16.7%, 25/150), nephrology (13.3%, 20/150), and other departments (16.7%, 25/150). Notably, Hospital B had a concentration of cases in the hepatobiliary-pancreatic surgery department, while Hospital E primarily reported cases from the burn unit.

### 3.2 The sensitivity of antimicrobial drugs

The MIC results obtained through instrumental methods and the broth microdilution method reveal that 150 CRKP strains had relatively high sensitivity to the basic drugs tigecycline, polymyxin B, and ceftazidime-avibactam. Additionally, the sensitivity to combination therapy drugs fosfomycin, amikacin, and chloramphenicol was also relatively high, with rates of 72, 40, and 16.7%, respectively. Furthermore, a certain proportion of the strains exhibited intermediate sensitivity to fosfomycin and chloramphenicol, at 9.3 and 8.7%, respectively. The resistance rates for the remaining drugs were all above 90% (Table 1).

**Table 1:** Resistance and sensitivity profiles of 150 CRKP strains to antimicrobial agents.

Antibacterial agents	Break point (MIC, μg/mL)		Sensitive		Intermediary		Resistance	
	R^a^	S^b^	N^c^	P^d^ (%)	N^c^	P^d^ (%)	N^c^	P^d^ (%)
Ertapenem	≥2	≤0.5	0	0.0	0	0.0	150	100.0
Imipenem	≥4	≤1	2	1.3	1	0.7	147	98.0
Levofloxacin	≥2	≤0.5	8	5.3	15	10.0	127	84.7
Piperacillin-Tazobactam	≥32/4	≤8/4	0	0.0	0	0.0	150	100.0
Ceftriaxone	≥4	≤1	0	0.0	0	0.0	150	100.0
Cefepime	≥32	≤8	0	0.0	1	0.7	149	99.3
SMZ-TMP	≥4/76	≤2/38	70	46.7	7	4.6	73	48.7
Ceftazidime	≥16	≤4	3	2.0	2	1.3	145	96.7
Amikacin	≥64	≤16	60	40.0	4	2.7	86	57.3
Amoxicillin-clavulanate	≥32/16	≤8/4	2	1.3	0	0.0	148	98.7
Fosfomycin	≥256	≤64	108	72.0	14	9.3	28	18.7
Aztreonam	≥16	≤4	13	8.7	1	0.6	136	90.7
Chloramphenicol	≥32	≤8	25	16.6	13	8.7	112	74.7
Tigecycline	≥8	≤2	148	98.7	2	1.3	0	0.0
Polymyxin B	>2	≤2	147	98.0	0	0.0	3	2.0
ceftazidime-avibactam	≥16/4	≤8/4	102	68.0	0	0.0	48	32.0

a Resistance breakpoint.

b Sensitivelass breakpoint.

c Number.

d Proportion.

### 3.3 The screening results for antibiotic resistance genes

**Figure 1:**
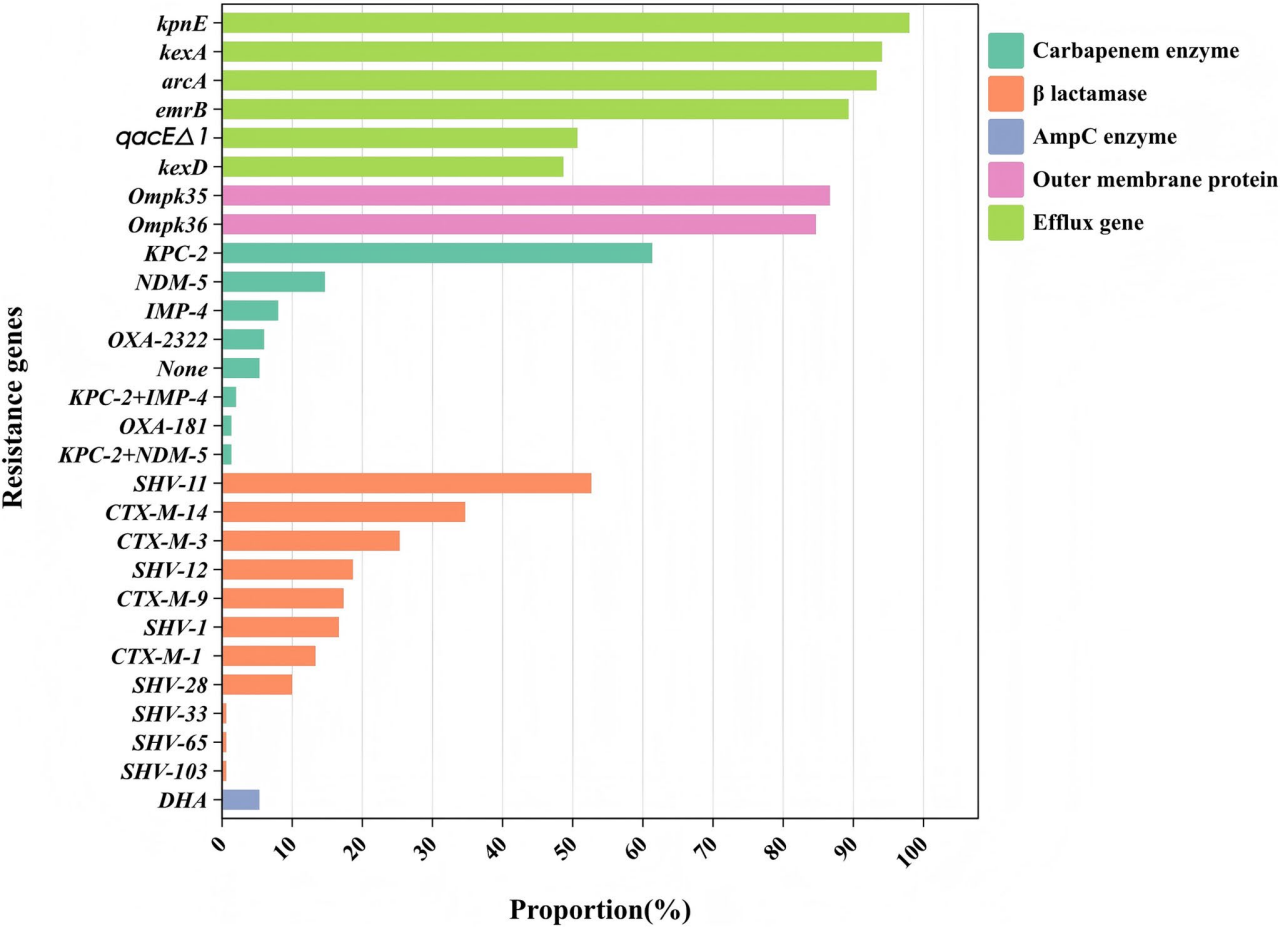
Classification of the resistance genes. Different colors represent different types of drug resistance genes.

**Figure 2:**
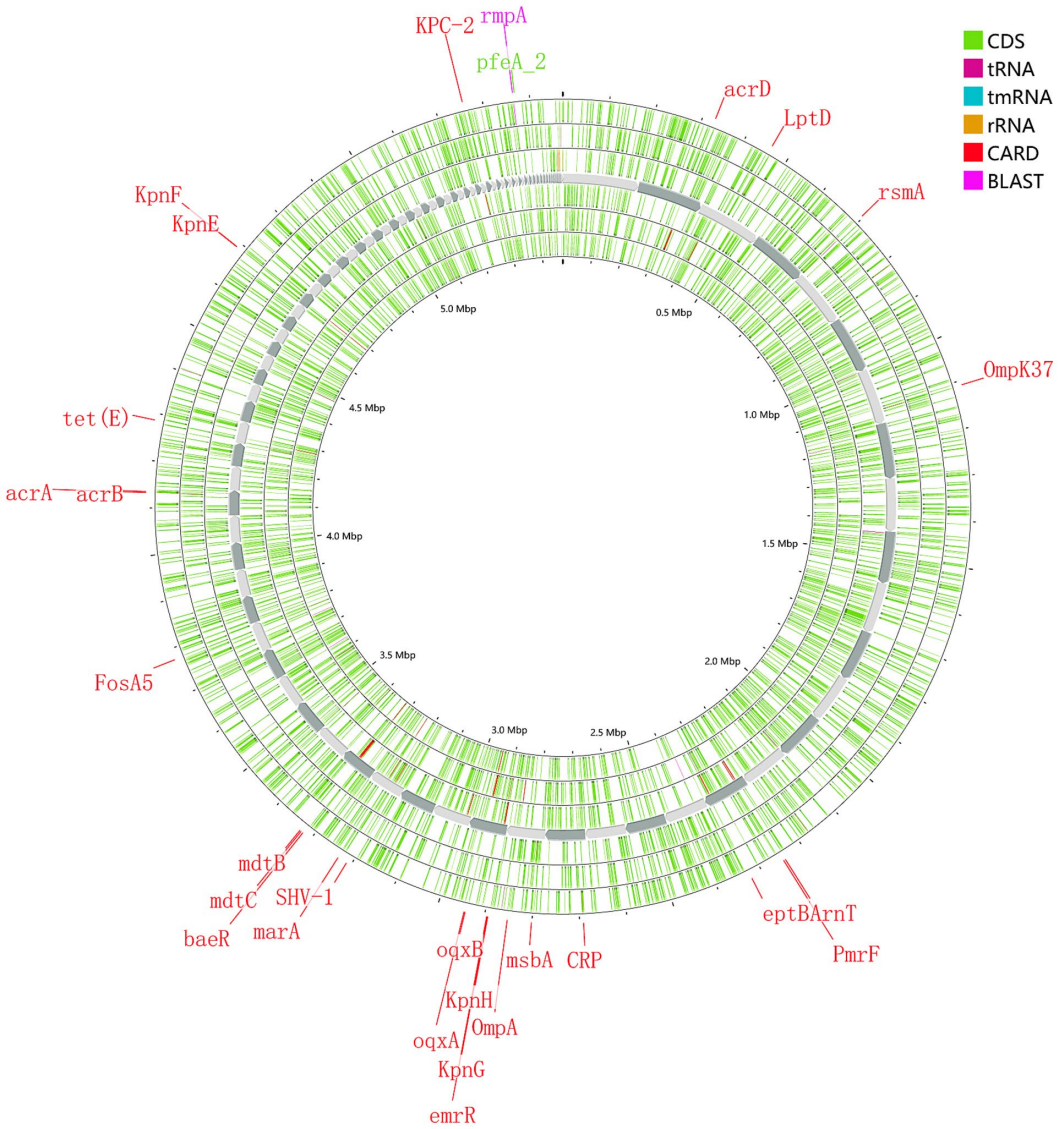
Whole genome sequencing of resistance and virulence genes of strain 111.

The main proportions of carbapemen genes producing in CRKP are as follows: *KPC-2* (61.3%), *NDM-5* (14.7%), *IMP-4* (8.0%), *OXA-232* (6.0%), and *OXA-181* (1.3%), respectively. No strains expressed *IMP-2, VIM, IMI, SME, GES, GIM, SIM, SPM, AIM*, or *DIM* genes were detected. The distribution of β-lactamase resistance genes in the analyzed isolates was as follows: *CTX-M-1* (13.33%), *CTX-M-3* (25.33%)*, CTX-M-9* (17.33%)*, CTX-M-14* (34.67%), and *SHV* (93.33%) were the most prevalent. Subsequent sequencing of the *SHV*-positive isolates revealed seven variants: *SHV-1* (16.66%)*, SHV-11* (52.66%)*, SHV-12* (18.66%)*, SHV-28* (10.00%), *SHV-65* (0.66%)*, SHV-103* (0.66%) and *SHV-33* (0.6%). For additional details on the distribution of other resistance genes, please refer to Figure 1. The genome of strain 111 (Figure 2) was selected for display because it carries *KPC-2*, the most prevalent carbapenemase gene in CRKP, and represents a comprehensive and typical profile of carbapenemase genes observed in CRKP. Strain 111 was chosen because it carries *KPC-2*, the most prevalent carbapenemase gene in CRKP. This combination of genes makes strain 111 highly representative of the resistance patterns observed in the studied population. Furthermore, the whole genome sequencing results of strain 111were consistent with the PCR-based detection of resistance genes, confirming the accuracy and reliability of the data. Its genomic profile not only reflects the dominant resistance mechanisms but also provides a clear example of the genetic diversity and complexity of CRKP strains.

### 3.4 Correlation analysis of the sensitivity of basic antimicrobial drugs and combination therapy drugs with different enzyme types

**Figure 3:**
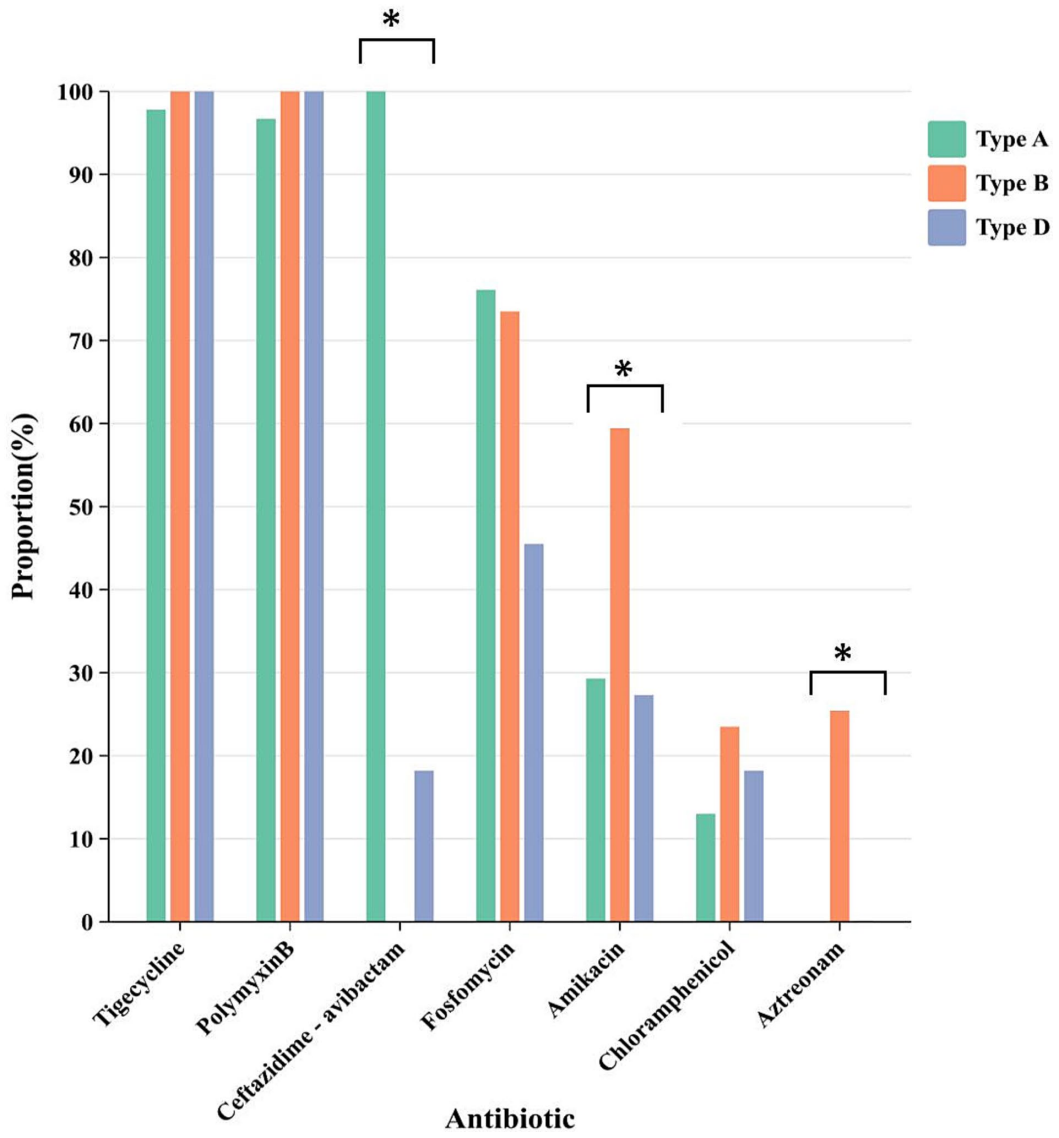
The differences in sensitivity of CRKP carrying different enzyme types to different drugs. Different colors represent different carbapenem enzyme types. * represents comparison of sensitivity rates, *p* < 0.01.

Analysis of the sensitivity of basic antimicrobial drugs in relation to the three main carbapenemase enzyme types revealed that CRKP producing class A, class B, and class D enzymes had sensitivity rates of 97.8, 100, and 100% to tigecycline, and 96.7, 100, and 100% to polymyxin B, respectively, indicating relatively high sensitivity rates. Specifically, CRKP producing class A enzymes exhibited a sensitivity rate of 100% to ceftazidime-avibactam, while CRKP producing class B and class D enzymes had sensitivity rates of only 0 and 18.2%, respectively (Figure 3). Ceftazidime-avibactam exhibited better inhibitory effects against CRKP strains producing class A enzymes (*p* < 0.01). The study on the differences in sensitivity to combination therapy drugs among the three main enzyme types of strains showed that the sensitivity rates of CRKP producing class A, class B, and class D enzymes to fosfomycin were 76.1, 73.5, and 45.5%, respectively. The sensitivity rates to amikacin were 29.3, 61.8, and 27.3%, respectively, and to chloramphenicol were 13, 23.5, and 18.2%, respectively. The sensitivity rates to aztreonam were 0.0, 29.4, and 0.1%, respectively. Aztreonam and amikacin exhibited a more significant inhibitory effect against CRKP producing class B enzymes (p < 0.01). The resistance rates with the remaining combination therapy drugs were relatively high, and no comparison of drug sensitivity rates was conducted (Figure 3).

### 3.5 The subtyping results for the MLST

**Figure 4:**
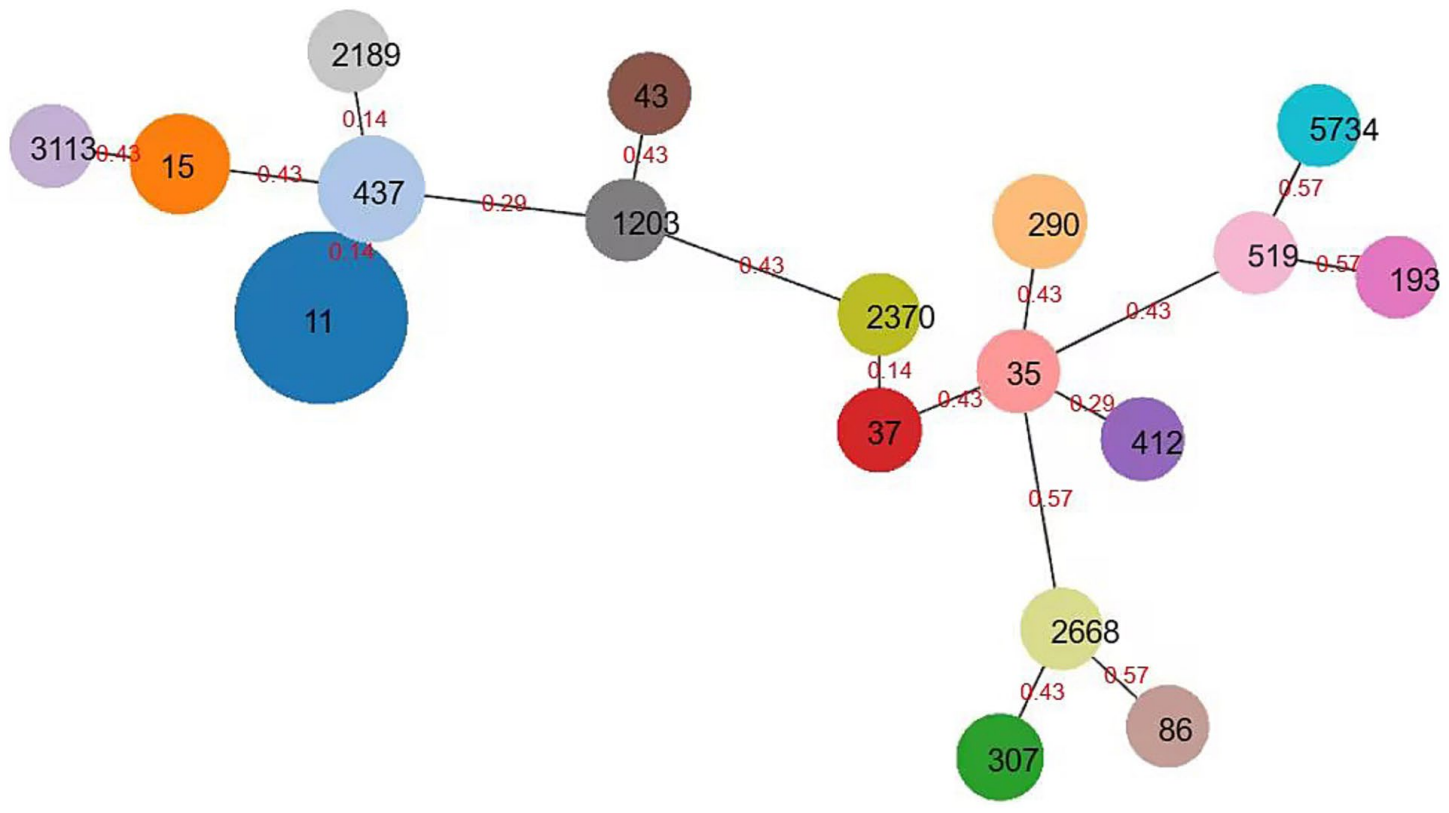
Minimum spanning tree of *Klebsiella pneumoniae*. The minimum spanning tree is constructed using seven allelic genes (gapA, infB, mdh, pgi, phoE, rpoB, tonB) of *Klebsiella pneumoniae*. The size of the nodes is proportional to the number of isolates, and the red number represents affinity, and the smaller the number, the closer the two ST types are.

According to the MLST typing method, the 150 CRKP were classified into 19 ST (sequence type) types. Among them, ST11 was the dominant clone (75/150, 50.00%), followed by ST437 (22/150, 14.67%), ST15 (16 strains, 10.67%), ST290 (11 strains, 7.33%), ST307 (5 strains, 3.33%), ST4 and ST37 (3 strains each, 2.00%), ST35, ST412, and ST3113 (2 strains each, 1.33%), and ST5734, ST86, ST519, ST2370, ST1203, ST2189, ST2668, ST43, and ST193 (1 strain each, 1/60, 1.67%). Among them, ST11, ST437, and ST2189 are phylogenetically related, while ST2370 and ST37 are also phylogenetically related. The minimum spanning tree is shown in Figure 4.

### 3.6 Difference in antibiotic resistance genes by different ST types

**Figure 5:**
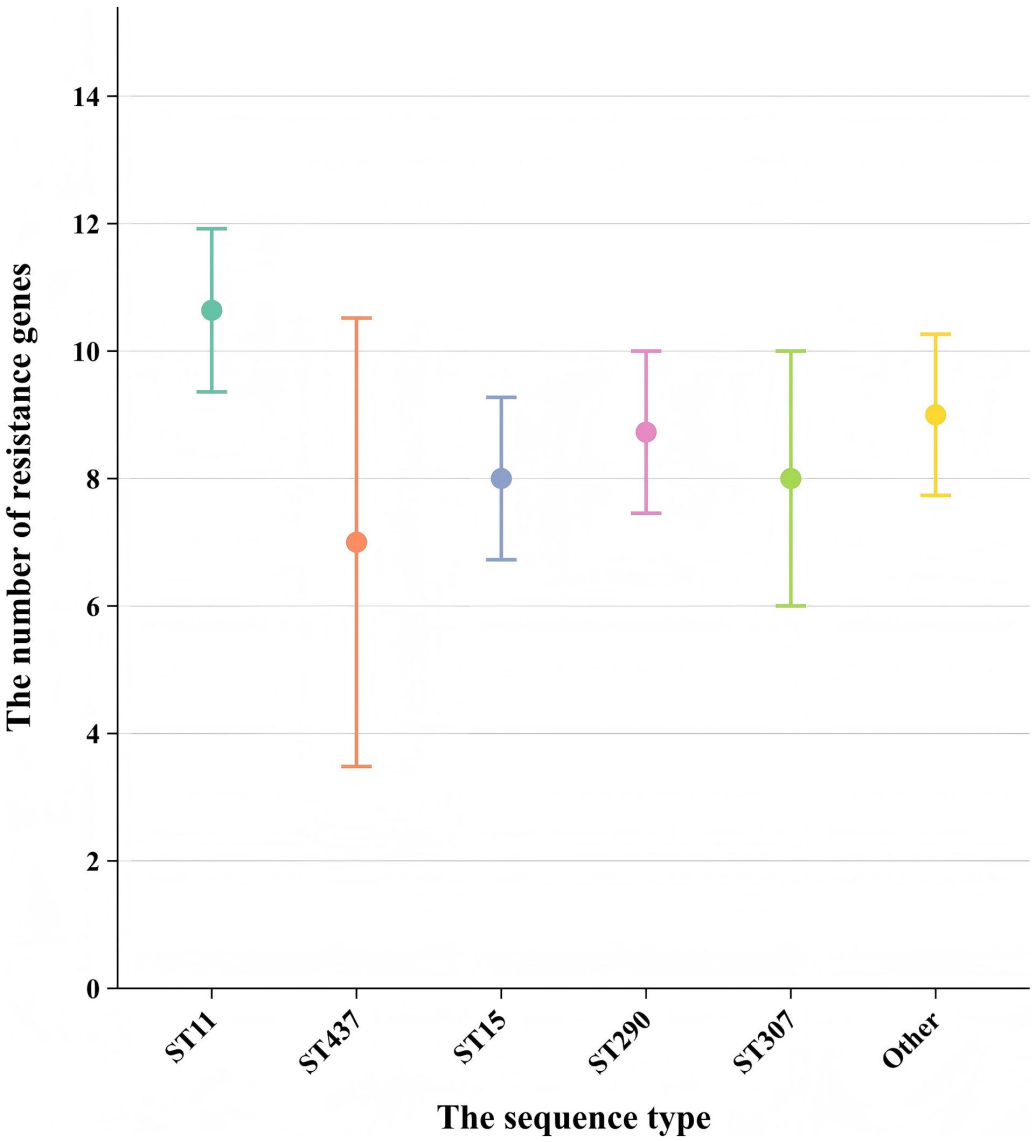
Comparison of the number of resistance genes carried among different sequence types (STs). Each point represents the mean number of resistance genes carried by strains of a specific ST. Error bars indicate the standard deviation (SD) derived from strains per ST. The x-axis shows the sequence type (ST), and the y-axis shows the number of resistance genes.

Analyzing the antibiotic resistance genes carried by different ST strains revealed that ST11 strains carry a higher number of resistance genes. Other strains, particularly those of ST437, exhibit a wide distribution in the number of resistance genes and, comparatively, carry fewer resistance genes (Figure 5). Comparison between ST11 and non-ST11 strains revealed that ST11 strains primarily carry the *KPC-2* carbapenemase gene, whereas other carbapenemase genes are predominantly carried by non-ST11 strains (*p* < 0.01). The presence of β-lactamase resistance genes *CTX-M-14*, *SHV-11*, and *SHV-12* types is mainly observed in ST11 strains, while the *CTX-M-1, SHV-1*, and *SHV-28* β-lactamase resistance gene is primarily found in non-ST11 strains (*p* < 0.05). Additionally, the presence of outer membrane protein genes *Ompk35* and *Ompk36* is predominantly associated with ST11 strains (*p* < 0.01). Similarly, the presence of efflux pump genes *arcA*, *kexD*, *kexA*, *emrB*, and *qacE△1* types is mainly observed in ST11 strains (*p* < 0.01), indicating statistically significant differences (Table 2).

**Table 2:** Comparison of drug resistance genes between ST11 type and non-ST11 type CRKP strains.

Resistance mechanism	Genotype	ST11 type (*n* = 75)	Non-ST11 type (*n* = 75)	χ^2^	*P*
Carbapenemase	*KPC-2*	70	22	67.768	<0.01
	*NDM-5*	0	22	25.781	<0.01
	*IMP-4*	1	11	9.058	<0.01
	*OXA-232*	0	9	9.574	<0.01
	*OXA-181*	0	2	2.027	0.16
	*KPC-2+NDM-1*	2	0	2.027	0.16
	*KPC-2+IMP-4*	2	1	0.340	0.56
β lactamase	*CTX-M-1*	2	18	14.769	<0.01
	*CTX-M-3*	16	22	1.269	0.26
	*CTX-M-9*	21	7	8.607	<0.01
	*CTX-M-14*	43	9	34.027	<0.01
	*SHV-1*	0	25	38.368	<0.01
	*SHV-11*	64	15	3.930	0.047
	*SHV-12*	28	0	10.465	0.0012
	*SHV-28*	0	15	55.4112	<0.01
AmpC enzyme	*DHA*	3	5	0.528	0.467
Outer membrane protein	*Ompk35*	75	55	23.077	<0.01
	*Ompk36*	71	56	11.554	<0.01
Efflux gene	*arcA*	75	65	10.714	<0.01
	*kexD*	56	17	40.589	<0.01
	*kexA*	75	66	9.574	<0.01
	*kpnE*	75	72	3.063	0.08
	*emrB*	74	60	13.713	<0.01
	*qacE△1*	54	22	27.312	<0.01

### 3.7 The distribution of carbapenemase genes and ST types in different medical institutions

**Figure 6:**
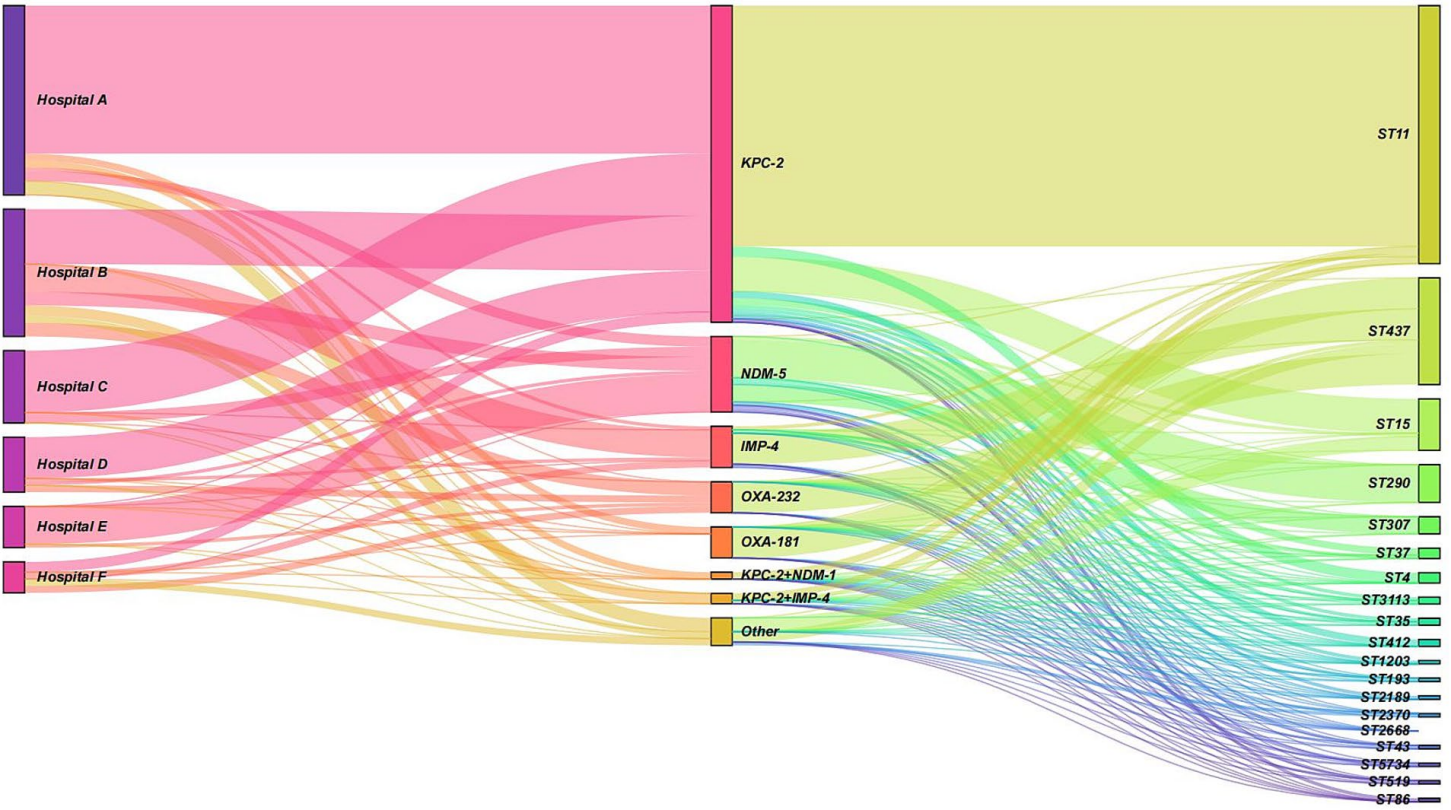
Sankey diagram illustrating the distribution of resistance genes and sequence types (STs) across hospitals. The diagram shows the flow and proportion of resistance genes (*KPC-2*, *NDM-5*, *IMP-4*, and *OXA-232*) and their associated ST types (e.g., ST11, ST15, ST290, ST307, and ST437) in hospitals A, B, C, D, and E. The width of the bands represents the relative proportion of isolates. *KPC-2* was the predominant gene in hospitals A, B, C, and D, while *NDM-5* was the most prevalent in hospital E. ST11 was the most common ST type among *KPC-2*-carrying isolates, whereas ST290 was the most frequent among *NDM-5*-carrying isolates. *IMP-4* and *OXA-232* were primarily associated with ST437.

In hospitals A, B, C, and D, the *KPC-2* gene predominated, accounted for 78.2, 43.3, 85.7, and 75.0%, respectively. Additionally, the *IMP-4* gene also constituted a significant proportion in hospital B, at 21.6%. In hospital E, the *NDM-5* gene was the most prevalent, accounted for 91.7%. There were notable differences in the distribution of ST types among different resistance gene types. Among the isolates carrying the *KPC-2* gene, the most common ST type was ST11 (*n* = 70, 76.1%), followed by ST15 (*n* = 10, 10.9%). In the *NDM-5* gene isolates, the most common ST type was ST290 (*n* = 11, 50.0%), followed by ST307 (*n* = 5, 22.7%). The *IMP-4* and *OXA-232* types were mainly associated with ST437. More details can be found in Figure 6.

## 4 Discussion

The emergence and rapid dissemination of carbapenem-resistant *Klebsiella pneumoniae* (CRKP) have become a global public health crisis, particularly in regions with high antibiotic consumption such as China (Hu et al., 2020; Ge et al., 2024). This study provides a comprehensive analysis of the antimicrobial susceptibility profiles, carbapenemase gene distribution, and molecular epidemiology of CRKP strains isolated from multiple hospitals in the Ningbo region. By integrating phenotypic and genotypic data, our findings not only elucidate the local resistance patterns but also offer valuable insights for optimizing treatment strategies and infection control measures in the context of global CRKP epidemiology.

The high resistance rates of CRKP to most commonly used antibiotics observed in our study are consistent with reports from other regions in China, such as Changsha (Jia et al., 2023) and Beijing (Wang et al., 2018), as well as globally (Jean et al., 2022). However, the relatively high sensitivity to tigecycline, polymyxin B, and ceftazidime-avibactam aligns with their recommended use as first-line treatments for CRKP infections (Zhuang et al., 2025). In comparison to other regions in China, the resistance rates observed in Ningbo are similar to those reported in Henan Province, where *KPC-2*-producing CRKP strains also exhibit high resistance to carbapenems but remain sensitive to polymyxin B and ceftazidime-avibactam (Wang et al., 2023). These differences may be attributed to variations in antibiotic usage patterns, infection control practices, and the prevalence of specific resistance mechanisms. Notably, the sensitivity of CRKP to combination therapy drugs such as fosfomycin, amikacin, and chloramphenicol was higher than expected, suggesting their potential utility in tailored treatment regimens. This contrasts with reports from some countries, where fosfomycin resistance rates are significantly higher due to its widespread use in urinary tract infections (Hurwitz et al., 2024). The regional differences highlight the importance of local antimicrobial resistance surveillance in guiding empirical therapy. Additionally, CRKP shows low resistance rates to levofloxacin and amikacin. The reason for this is that in clinical practice, these three drugs are typically not used individually but rather as components of combination therapy. This approach can enhance treatment efficacy and mitigate the development of bacterial resistance to medications. The lower resistance rates to SMZ-TMP compared to β-lactam antibiotics can be attributed to their different mechanisms of action (Li et al., 2020). However, while SMZ-TMP demonstrates sensitivity *in vitro*, its efficacy *in vivo* may be limited.

CRKP exhibits a complex mechanism of resistance to carbapenem antibiotics, with the most common mechanism being the production of carbapenemases. The *KPC-2* gene was predominant in hospitals A, C, and D (78.2, 85.7, and 75.0%, respectively), consistent with its high prevalence in eastern China (Kong et al., 2020; Li et al., 2025). *NDM* and *VIM* are more prevalent in South Asia and Europe, respectively (Gajdács et al., 2020). For example, in India, *NDM* is the dominant carbapenemase gene, accounting for over 70% of CRKP isolates (Kumarasamy et al., 2010), while in Poland, *VIM* is the most common carbapenemase gene (Izdebski et al., 2023). However, the high proportion of *IMP-4* (21.6%) in hospital B and *NDM-1* (91.7%) in hospital E suggests localized outbreaks driven by specific resistance genes. The occurrence of CRKP strains with identical resistance genes across different hospitals or departments may be attributed to nosocomial cross-transmission, patient referrals, or healthcare worker-mediated spread. Furthermore, factors such as antibiotic prescribing practices, environmental contamination levels, and the frequency of invasive procedures can significantly influence bacterial colonization and dissemination, leading to the clustering of resistant strains in high-risk units. These findings underscore the importance of tailored infection control strategies that consider the unique risks and patient populations in different hospital departments. Enhanced surveillance, strict adherence to hand hygiene, and targeted decolonization efforts may help mitigate the spread of CRKP within high-risk departments. Additionally, further research into the molecular epidemiology of CRKP strains within specific departments could provide valuable insights into their transmission dynamics and inform more effective intervention strategies. The co-occurrence of *KPC-2* and *NDM-1* genes in some strains is particularly concerning, as it may limit treatment options and facilitate the spread of multidrug resistance. In our study, carrying both *KPC-2* and *NDM-1* types, as well as *KPC-2* and *IMP-4* types, were found. This phenomenon has also been reported in other regions, such as India and the Middle East, where the co-production of multiple carbapenemases is increasingly common (Kumarasamy et al., 2010). These regional differences highlight the importance of tailoring infection control strategies to local epidemiological patterns. Further investigation into other patient information revealed that patients infected with CRKP are mainly concentrated in the elderly population aged 55 years and older, who generally have relatively weakened immune systems and are more susceptible to superbug infections. However, the gender distribution is relatively balanced. Analysis indicates that there is no significant correlation between enzyme type distribution and patient age or gender.

Strains carrying different β-lactamase enzyme types (class A, B, and D) exhibit distinct resistance profiles, leading to varying sensitivities to antimicrobial agents and influencing therapeutic decisions (da Costa de Souza et al., 2022). In this study, CRKP carrying class A, B, and D enzymes exhibited high sensitivity to tigecycline and polymyxin B, consistent with previous reports highlighting these drugs as last-resort options against carbapenem-resistant Enterobacterales (CRE) (Chang et al., 2022). Regarding ceftazidime-avibactam (CZA) susceptibility, CRKP strains harboring class A enzymes (particularly KPC) showed a sensitivity rate of 100%, aligning with studies confirming CZA’s potent activity against KPC-producing isolates (Huang et al., 2021). In contrast, strains carrying class B and class D enzymes exhibited significantly reduced susceptibility, corroborating findings that avibactam does not inhibit metallo-β-lactamases (Xiong et al., 2022). For metalloenzyme-producing CRKP (class B), combination therapies involving aztreonam and amikacin demonstrated notable inhibition zones, likely due to aztreonam’s stability against metallo-β-lactamases despite its susceptibility to serine β-lactamases (Vázquez-Ucha et al., 2023). This observation aligns with clinical studies advocating aztreonam-avibactam combinations for NDM-producing Enterobacterales (Delp et al., 2024). However, other tested combinations (e.g., ceftazidime, amoxicillin/clavulanic acid, cefepime, and rifampin) showed limited efficacy despite statistically significant differences in zone diameters, underscoring the need for tailored regimens based on enzyme type.

In addition to carbapenemase production, the loss of outer membrane proteins (e.g., *Ompk35* and *Ompk36*) and the overexpression of efflux pumps (e.g., *arcA*, *kexD*, *emrB*) were identified as key resistance mechanisms in CRKP strains. These mechanisms contribute to reduced antibiotic penetration and increased drug efflux, further complicating treatment (Alenazy, 2022; Onishi et al., 2022). The widespread distribution of *CTX-M* β-lactamase genes among CRKP strains also underscores their high resistance to β-lactam antibiotics, necessitating the use of alternative therapeutic strategies. In this study, a certain proportion of strains exhibited loss of the outer membrane protein genes *Ompk35* and *Ompk36*, with the loss rates of these genes being 13.4 and 15.4%, respectively. The loss of these outer membrane proteins can compromise the permeability of the bacterial cell wall, thereby contributing to increased antibiotic resistance. Furthermore, efflux pumps play a critical role in antibiotic resistance by actively transporting drugs from inside the cell to the outside, reducing the effective drug concentration and enhancing the resistance of the cell to drugs (Alenazy, 2022). In this study, a significant proportion of strains were found to carry efflux pump genes such as *arcA*, *kexD*, *kexA*, *kpnE*, *emrB*, and *qacE△1*. This enhances CRKP’s ability to actively pump out antibiotics, thereby further increasing antibiotic resistance.

Multilocus sequence typing (MLST) revealed that ST11 was the dominant sequence type (50.0%) among CRKP strains in the Ningbo region, consistent with reports from other parts of China (Liao et al., 2020). The ST11 strains have broad dissemination capability and adaptability, enabling them to survive and proliferate in diverse environments. This makes them more prone to acquiring and disseminating resistance genes, leading to their high revalence in the local area. In addition, our study results further demonstrate molecular characteristic differences among different ST types of strains. For example, ST11 strains exhibited a higher prevalence of resistance genes, including *KPC-2*, *CTX-M-9*, *CTX-M-14*, and *SHV-11/SHV-12* (ESBL variants), compared to non-ST11 strains. In contrast, non-ST11 strains were more likely to carry *NDM-5* and *IMP-4* carbapenemase genes, as well as *CTX-M-1* and non-ESBL *SHV-1/SHV-28* β-lactamase genes (Talebzadeh et al., 2022). The predominance of *SHV-11* and *SHV-12* (both confirmed ESBLs) in ST11 strains suggests a lineage-specific adaptation favoring extended-spectrum resistance, whereas non-ST11 strains predominantly harbored *SHV-1* (a narrow-spectrum β-lactamase) and *SHV-28* (a rare variant with uncertain ESBL phenotype). This divergence underscores the role of ST11 in propagating ESBL-associated resistance, potentially due to plasmid compatibility or selective pressures in clinical environments. These findings suggest that ST11 strains have a greater capacity for acquiring and disseminating resistance genes, contributing to their widespread prevalence. The high genetic homogeneity of ST11 strains within individual hospitals indicates potential intra-hospital transmission, likely facilitated by the movement of healthcare workers, contaminated medical equipment, and environmental surfaces. This is consistent with reports from other regions (Li et al., 2022).

In contrast, ST258 remains the most common sequence type in Western countries, highlighting the regional variability in CRKP epidemiology (Unlu et al., 2021). For example, in the United States, ST258 strains are responsible for the majority of CRKP infections, particularly in intensive care units (Chen et al., 2014). The genetic similarity between ST11 and ST258 suggests a possible evolutionary relationship, although further studies are needed to elucidate their origins and dissemination patterns. Other ST types may also cause outbreaks, necessitating further research, detection, and control measures for these clones. In addition, strong association of *KPC-2* with ST11 (76.1%) and *NDM-5* with ST290 (50.0%) highlights the role of high-risk clones in disseminating resistance. These findings underscore the need for tailored infection control measures. Hospitals with high *KPC-2*-producing ST11 strains should prioritize strict antibiotic stewardship, while those with *NDM-5*-producing ST290 or *IMP-4*-producing ST437 strains may require targeted interventions, such as screening high-risk patients and enhancing contact precautions. Continuous surveillance is essential to monitor emerging resistance patterns and prevent the spread of multidrug-resistant clones.

Our findings have several clinical implications. First, the high sensitivity of CRKP to tigecycline, polymyxin B, and ceftazidime-avibactam supports their continued use as first-line treatments. However, the potential for heteroresistance and toxicity associated with these drugs necessitates careful monitoring and dose optimization (Ma et al., 2019; Fang et al., 2023). Second, the observed sensitivity of CRKP to combination therapy drugs such as fosfomycin and amikacin suggests their potential utility in tailored treatment regimens. Finally, the identification of hospital-specific resistance patterns underscores the need for targeted infection control measures to prevent the spread of CRKP within healthcare facilities. Future studies should focus on longitudinal surveillance of CRKP strains to monitor emerging resistance patterns and evaluate the effectiveness of intervention strategies. The integration of epidemiological data with molecular insights will be crucial for addressing the global challenge of CRKP infections.

## 5 Conclusion

In summary, CRKP showed high sensitivity to tigecycline, polymyxin B, ceftazidime-avibactam, fosfomycin, amikacin, and chloramphenicol. The main carbapenemase genes identified were *KPC-2* and *NDM-5*. The inhibitory effects of ceftazidime-avibactam, aztreonam, and amikacin varied for CRKP carrying different enzyme types. ST11 strains were predominant in the region. There was a significant difference in the resistance genes carried by ST11 strains compared to non-ST11 strains. Clonal dissemination was observed both within the same healthcare institution and between different institutions.

## Data Availability

The complete genome sequences of 47 strains of CRKP were deposited in GenBank with accession number PRJNA1241480.

## Footnotes

^1^ http://www.carss.cn/

^2^Available at: https://bigsdb.pasteur.fr/cgi-bin/bigsdb/bigsdb.pl?db=pubmlst_klebsiella_seqdef.

^3^ http://www.phyloviz.net/
