# Supplementary material for: Molecular characterization of carbapenem resistance mechanisms and phenotypic correlations in clinical Klebsiella pneumoniae isolates from Ningbo, China
Source: Front Microbiol. 2025 May 14;16:1546805. doi: 10.3389/fmicb.2025.1546805 (PMC12116675; doi:10.3389/fmicb.2025.1546805)
Supplement: Supplementary file 1 [file Table_1.docx]

**Supplemental material**

**Supplementary Table1** Primers used for PCR amplification

| **Primer** | **Primer sequence(5＇-3＇)** | **Product**  **（bp）** | **Reference** |
| --- | --- | --- | --- |
| *KPC-2-F* | ATGTCACTGTATCGCCGTCT | 920 | (Liu et al., 2021) |
| *KPC-2-R* | TTTTCAGAGCCTTACTGCCC |  |  |
| *IMP-1-F* | CATGGTTTGGTGGTTCTTGT | 526 | (Liu et al., 2021) |
| *IMP-1-R* | GTAMGTTTCAAGAGTGATGC |  |  |
| *IMP-2-F* | GGCAGTCGCCCTAAAACAAA | 737 | (Martirosov and Lodise, 2016) |
| *IMP-2-R* | TAGTTACTTGGCTGTGATGG |  |  |
| *VIM-F* | GTTTGGTCGCATATCGCAAC | 645 | (Liu et al., 2021) |
| *VIM-R* | CTACTCGGCGACTGAGCGAT |  |  |
| *NDM-1-F* | CAGCACACTTCCTATCTC | 292 | (Liu et al., 2021) |
| *NDM-1-R* | CCGCAACCATCCCCTCTT |  |  |
| *OXA-181-F* | GCTTGATCGCCCTCGATT | 438 | This study |
| *OXA-181-R* | GCCCCTCTGCCCGAAAGT |  |  |
| *OXA-232-F* | GGTTGTAGCGGTTGATCG | 438 | This study |
| *OXA-232-R* | CAGCACTTCTTTTGTGATGGC |  |  |
| *IMI-F* | CCATTCACCCATCACAAC | 440 | (Moghadampour et al., 2018) |
| *IMI-R* | CTACCGCATAATCATTTGC |  |  |
| *SME-F* | ACTTTGATGGGAGGATTGGC | 551 | (Candevir Ulu et al., 2017) |
| *SME-R* | ACGAATTCGAGATCACCAG |  |  |
| *GES-F* | GTTTTGCAATGTGCTCAACG | 371 | (Liu et al., 2021) |
| *GES-R* | TGCCATAGCAATAGGCGTAG |  |  |
| *GIM-F* | AGAACCTTGACCGAACGCAG | 746 | (Liu et al., 2021) |
| *GIM-R* | ACTCATGACTCCTCACGAGG |  |  |
| *SIM-F* | TACAAGGGATTCGGCATCG | 571 | (Liu et al., 2021) |
| *SIM-R* | TAATGGCCTGTTCCCATGTG |  |  |
| *SPM-F* | GCGTTTTGTTTGTTGCTC | 786 | (Liu et al., 2021) |
| *SPM-R* | TTGGGGATGTGAGACTAC |  |  |
| *CMY-F* | TGGCCAGAACTGACAGGCAAA | 462 | (Shi et al., 2022) |
| *CMY-R* | TTTCTCCTGAACGTGGCTGGC |  |  |
| *AIM-F* | CTGAAGGTGTACGGAAACAC | 322 | (Chen et al., 2022) |
| *AIM-R* | GTTCGGCCACCTCGAATTG |  |  |
| *DIM-F* | GCTTGTCTTCGCTTGCTAACG | 699 | (Chen et al., 2022) |
| *DIM-R* | CGTTCGGCTGGATTGATTTG |  |  |
| *DHA-F* | CTGATGAAAAAATCGTTATC | 898 | (Liu et al., 2021) |
| *DHA-R* | ATTCCAGTGCACTCAAAATA |  |  |
| *CIT-F* | TGGCCAGAACTGACAGGCAAA | 462 | (Satter et al., 2020) |
| *CIT-R* | TTTCTCCTGAACGTGGCTGGC |  |  |
| *EBC-F* | TCGGTAAAGCCGATGTTGCGG | 302 | (Satter et al., 2020) |
| *EBC-R* | CTTCCACTGCGGCTGCCAGTT |  |  |

**Supplementary Table1（Cont）**

| **Primer** | **Primer sequence(5＇-3＇)** | **Product**  **（bp）** | **Reference** |
| --- | --- | --- | --- |
| *MOX-F* | GCTGCTCAAGGAGCACAGGAT | 520 | (Satter et al., 2020) |
| *MOX-R* | CACATTGACATAGGTGTGGTGC |  |  |
| *ACC-F* | AACAGCCTCAGCAGCCGGTTA | 346 | (Satter et al., 2020) |
| *ACC-R* | TTCGCCGCAATCATCCCTAGC |  |  |
| *FOX-F* | AACATGGGGTATCAGGGAGATG | 190 | (Satter et al., 2020) |
| *FOX-R* | CAAAGCGCGTAACCGGATTGG |  |  |
| *CTX-M-1-F* | GGCCCATGGTTAAAAAATCACTGC | 944 | (Lahlaoui et al., 2014) |
| *CTX-M-1-R* | CAGCGCTTTTGCCGTCTAAG |  |  |
| *CTX-M-2-F* | ATGATGACTCAGAGCATTCG | 876 | (Lahlaoui et al., 2014) |
| *CTX-M-2-R* | TTATTGCATCAGAAACCGTG |  |  |
| *CTX-M-3-F* | GTTGTTGTTATTTCGTATCTTCC | 934 | (Lahlaoui et al., 2014) |
| *CTX-M-3-R* | CGATAAACAAAAACGGAATG |  |  |
| *CTX-M-8-F* | ATGATGAGACATCGCGTTAAG | 864 | (Lahlaoui et al., 2014) |
| *CTX-M-8-R* | CGGTGACGATTTTCGCGGCAG |  |  |
| *CTX-M-9-F* | GTTACAGCCCTTCGGCGATGATTC | 898 | (Lahlaoui et al., 2014) |
| *CTX-M-9-R* | GCGCATGGTGACAAAGAGAGTGCAA |  |  |
| *CTX-M-10-F* | GCAGCACCAGTAAAGTGATGG | 873 | (Lahlaoui et al., 2014) |
| *CTX-M-10-R* | GCGATATCGTTGGTGGTACC |  |  |
| *CTX-M-14-F* | ACAATGACGCTGGCAGAACTG | 512 | (Lahlaoui et al., 2014) |
| *CTX-M-14-R* | TTACAGCCCTTCGGCGATGA |  |  |
| *CTX-M-25-F* | CACACGAATTGAATGTTCAG | 914 | (Lahlaoui et al., 2014) |
| *CTX-M-25-R* | TCACTCCACATGGTGAGT |  |  |
| *TEM-F* | TCGGGGAAATGTGCG | 877 | (Liu et al., 2021) |
| *TEM-R* | TGCTTAATCAGTGAGGCACC |  |  |
| *SHV-F* | GCCTTTATCGGCCTTCACTCAAG | 972 | (Liu et al., 2021) |
| *SHV-R* | TTAGCGTTGCCAGTGCTCGATCA |  |  |
| *OmpK35-F* | GGATGGAAAGATGCCTTCAG | 1392 | (Sugawara et al., 2016) |
| *OmpK35-R* | CATGACGAGGTTCCATTGTG |  |  |
| *OmpK36 -F* | GGGAAGAATCGCACGAAATA | 1744 | (Sugawara et al., 2016) |
| *OmpK36 -R* | TCTTACCAGGGCGACAAGAG |  |  |
| *acrA-F* | GTCCTCAGGTCAGTGGCATTA | 257 | (Türkel et al., 2018) |
| *acrA-R* | ATTGCTCTGCTGCGCCGTT |  |  |
| *oqxB-F* | TCATTGGCGGCGTGAAGA | 165 | (Türkel et al., 2018) |
| *oqxB-R* | CGGCGTGTTGGTGAACTGC |  |  |
| *kexD-F* | ACCGGTTGCGCAATACCCTGA | 184 | (Türkel et al., 2018) |
| *kexD-R* | CGTAATTGACGCCATCCCTG |  |  |
| *kdeA-F* | GTTGYYCCCGTTATGTCTGGTGC | 170 | (Türkel et al., 2018) |
| *kdeA-R* | CCAGCAGCCACTGTAAAAACATGC |  |  |
| *kpnE-F* | ATTGCTGAAATTACCGGCAC | 172 | (Türkel et al., 2018) |
| *kpnE-R* | AAATACCGATCCCTTCCCAC |  |  |

**Supplementary Table1（Cont）**

| **Primer** | **Primer sequence(5＇-3＇)** |  | **Reference** |
| --- | --- | --- | --- |
| *emrB-F* | CGACATTCATGCAGGTGCT | 848 | (Türkel et al., 2018) |
| *emrB-R* | GGTCGCCGTGTAACCGTAG |  |  |
| *oqxA-F* | CCGCTAAGGTGCTGGTGAAG | 933 | (Türkel et al., 2018) |
| *oqxA-R* | CCTTCTGAACGATGCGTAAACC |  |  |
| *rpoB-F* | GTTTTCCCAGTCACGACGTTGTAGGCGAAATGGCCGAGAACCA | 501 | (Liu et al., 2021) |
| *rpoB-R* | TTGTGAGCGGATAACAATTTCGAGTCTTCGAAGTTGTAACC |  |  |
| *gapA-F* | GTTTTCCCAGTCACGACGTTGTATGAAATATGACTCCACTCACGG | 450 | (Liu et al., 2021) |
| *gapA-R* | TTGTGAGCGGATAACAATTTCCTTCAGAAGCGGCTTTGATGGCTT |  |  |
| *mdh-F* | GTTTTCCCAGTCACGACGTrGTACCCAACTCGCTTCAGGTTCAG | 477 | (Liu et al., 2021) |
| *mdh-R* | TTCTGAGCGGATAACAATTTCCCGTTTTTCCCCAGCAGCAG |  |  |
| *pgi-F* | GTTTTCCCAGTCACGACGTTGTAGAGAAAAACCTGCCTGTACTGCTGC | 432 | (Liu et al., 2021) |
| *pgi-R* | TTGTGAGCGGATAACAATTTCCGCGCCACGCTTTATAGCGGTTAAT |  |  |
| *phoE-F* | GTTTTCCCAGTCACGACGTTGTAACCTACCGCAACACCGACTTCTTCGG | 420 | (Liu et al., 2021) |
| *phoE-R* | TTGTGAGCGGATAACAATTTCTGATCAGAACTGGTAGGTGAT |  |  |
| *infB-F* | GTTTTCCCAGTCACGACGTTGTACTCGCTGCTGGACTATATTCG | 318 | (Liu et al., 2021) |
| *infB-R* | TTGTGAGCGGATAACAATTtCCGCTTTCAGCTCAAGAACTTC |  |  |
| *tonB-F* | GTTTTCCCAGTCACGACGTTGTACTTTATACCTCGGTACATCAGGTT | 414 | (Liu et al., 2021) |
| *tonB-R* | TTGTGAGCGGATAACAATTTCATTCGCCGGCTGCGCGGAGAG |  |  |

**References**

Candevir Ulu, A., Güven Gökmen, T., Kibar, F., Kurtaran, B., Önlen, C., Kuşçu, F., et al. (2017). Molecular epidemiology of carbapenem-resistant Klebsiella pneumoniae at a Turkish centre: Is the increase of resistance a threat for Europe? J Glob Antimicrob Resist 11, 10-16. doi:10.1016/j.jgar.2017.06.012.

Chen, D., Xiao, L., Hong, D., Zhao, Y., Hu, X., Shi, S., et al. (2022). Epidemiology of resistance of carbapenemase-producing Klebsiella pneumoniae to ceftazidime-avibactam in a Chinese hospital. J Appl Microbiol 132, 237-243. doi:10.1111/jam.15166.

Lahlaoui, H., Ben Haj Khalifa, A., and Ben Moussa, M. (2014). Epidemiology of Enterobacteriaceae producing CTX-M type extended spectrum β-lactamase (ESBL). Med Mal Infect 44, 400-404. doi:10.1016/j.medmal.2014.03.010.

Liu, S., Wang, X., Ge, J., Wu, X., Zhao, Q., Li, Y.M., et al. (2021).

. Evid Based Complement Alternat Med 2021, 3455121. doi:10.1155/2021/3455121.

Martirosov, D.M., and Lodise, T.P. (2016). Emerging trends in epidemiology and management of infections caused by carbapenem-resistant Enterobacteriaceae. Diagn Microbiol Infect Dis 85, 266-275. doi:10.1016/j.diagmicrobio.2015.10.008.

Moghadampour, M., Salari-Jazi, A., and Faghri, J. (2018). High rate of carbapenem-resistant Klebsiella pneumoniae detected from hospital equipments in Iran. Acta Microbiol Immunol Hung 65, 529-538. doi:10.1556/030.65.2018.039.

Satter, S., Mahbub, H., and Shamsuzzaman, S.M. (2020). Phenotypic and Molecular Characterization of AmpC beta-lactamase Enzyme Producing Escherichia coli and Klebsiella species Isolated from A Tertiary Care Hospital in Bangladesh. Mymensingh Med J 29, 895-900.

Shi, Q., Han, X., Huang, Q., Meng, Y., Zhang, P., Wang, Z., et al. (2022). The Genetic Characteristics and Carbapenem Resistance Mechanism of ST307 Klebsiella pneumoniae Coharbouring bla(CMY-6), bla(OXA-48), and a Truncated bla(NDM-1). Antibiotics (Basel) 11. doi:10.3390/antibiotics11111616.

Sugawara, E., Kojima, S., and Nikaido, H. (2016). Klebsiella pneumoniae Major Porins OmpK35 and OmpK36 Allow More Efficient Diffusion of β-Lactams than Their Escherichia coli Homologs OmpF and OmpC. J Bacteriol 198, 3200-3208. doi:10.1128/jb.00590-16.

Türkel, İ., Yıldırım, T., Yazgan, B., Bilgin, M., and Başbulut, E. (2018). Relationship between antibiotic resistance, efflux pumps, and biofilm formation in extended-spectrum β-lactamase producing Klebsiella pneumoniae. J Chemother 30, 354-363. doi:10.1080/1120009x.2018.1521773.
